# The Type of Fat Ingested at Breakfast Influences the Plasma Lipid Profile of Postmenopausal Women

**DOI:** 10.1155/2014/815915

**Published:** 2014-07-21

**Authors:** J. M. Morillas-Ruiz, J. M. Delgado-Alarcon, J. M. Rubio-Perez, M. D. Albaladejo Oton

**Affiliations:** ^1^Department of Food Technology and Nutrition, Catholic University San Antonio of Murcia, Campus Los Jeronimos, Guadalupe, 30107 Murcia, Spain; ^2^Service of Biochemistry, Hospital Universitario Santa Lucia of Cartagena, Murcia, Spain

## Abstract

To assess whether the type of fat ingested at breakfast can modify the plasma lipid profile and other cardiovascular risk variables in postmenopausal women at risk of cardiovascular disease, a longitudinal, randomized, and crossover study was carried out with postmenopausal women at risk of CVD. They were randomly assigned to eat each type of breakfast during one month: 6 study periods (breakfast with the same composition plus butter/margarine/virgin olive oil) separated by two washout periods. On the first and last days of each study period, weight, arterial blood pressure, heart rate, and body mass index were recorded in fasting conditions and a blood sample was collected to measure plasma lipid profile. When comparing final values to baseline values, we only found out statistically significant differences on plasma lipid profiles. Butter-based breakfast increased total cholesterol and HDL, while margarine-based breakfast decreased total cholesterol and LDL and increased HDL. After the olive oil-based breakfast intake, a tendency towards a decrease of total cholesterol and LDL levels and an increase of HDL levels was observed. No statistically significant differences were observed in triglycerides levels, BMI, and arterial pressure in any breakfast type. The margarine-based breakfast was the only one which significantly increased the percentage of volunteers with optimal lipid profiles. The polyunsaturated fat at breakfast has improved the plasma lipid profile in the analyzed sample population, suggesting that PUFA-based breakfast can be advisable in women at risk of CVD.

## 1. Introduction

There is consistent evidence that the intake of different fatty acids through diet can modify plasma lipid profile and, therefore, CVD [1–5]. Nevertheless, there are no studies which analyze whether the type of fat consumed at breakfast could change the plasma lipid profile.

Gender (female), physiological-age (postmenopausal), and obesity (defined as a BMI >  30 kg/m^2^) are also risk factors related to the development of CVD [6–8]. The prevalence of these risk factors in the developed countries has been doubled from 1960 until now. Moreover, during the same period of time, although there is an inverse relationship between BMI and having breakfast, [9, 10] the number of adults who do not have breakfast has been almost doubled, due to different reasons such as lack of time or overweight [11]. On the other hand, it is also well known that the type of food ingested at breakfast influences BMI [12, 13]. In relation to this, a study carried out by González-Ortiz et al. [14] with healthy subjects showed that a daily fat-enriched breakfast intake significantly increased triglycerides plasma levels and BMI, in comparison with a carbohydrate-enriched breakfast. Furthermore, Pasman et al. [15], in a crossover study, showed that the consumption of a breakfast containing simple carbohydrates resulted, after 30 minutes, in higher glucose, insulin, triglycerides, and free fatty acids levels than after a breakfast containing complex carbohydrates.

While there is a wide range of studies which suggest that the quantitative and qualitative composition of food ingested at breakfast could influence plasma biochemical parameters, there is still a lack of studies which have specifically assessed the intake effect of different types of fat at breakfast on plasma lipid profile.

In this context, the aim of this study was to assess the effect of three types of fat (SFA, PUFA, and MUFA) ingested at breakfast (as butter, margarine, and olive oil, resp.) on plasma levels of total cholesterol, LDL, HDL, triglycerides, and other cardiovascular risk variables (BMI and arterial pression) in postmenopausal women at risk of CVD.

## 2. Material and Methods

### 2.1. Study Population

Sixty Caucasian white senior postmenopausal women and resident in Murcia (Spain) were recruited, of whom 53 (88%) completed the study (evaluable population). All volunteers fulfilling the inclusion criteria were enrolled in the study after the formal signature of their informed own consent. The study was performed following the principles of Helsinki declaration and the protocol was approved by the Ethic Committee of Catholic University San Antonio from Murcia (Spain).

### 2.2. Experimental Design

This was a longitudinal, randomized, and crossover clinical trial designed to evaluate the effect of the intake of three types of fat, as part of a breakfast, on plasma biochemical parameters and anthropometric variables. Breakfast A contained 20 g of butter (SFA source), breakfast B contained 20 g of margarine (PUFA source), and breakfast C contained 20 g of virgin olive oil (MUFA source). The fatty acid composition of butter was 81.1 g fat/100 g food [51.4 g SFA (butyric acid 3.2 g, caproic acid 2 g, caprylic acid 1.2 g, capric acid 2.5 g, lauric acid 2.6 g, myristic acid 7.4 g, palmitic acid 21,7 g, and stearic 9.9 g), 21 g MUFA (palmitoleic acid 0.96 g, oleic acid 19.9 g), 3 g PUFA (linoleic acid 2.7 g, linolenic acid 0.3 g), and 215 mg of cholesterol]. The fatty acid margarine profile was 60 g fat/100 g food[12.8 g SFA (caprylic acid 0.1 g, capric acid 0.1 g, lauric acid 1.7 g, myristic acid 0.6 g, palmitic acid 5.6 g, and stearic 4.2 g), 15.3 g MUFA (oleic acid 15.3 g), 29.2 g PUFA (linoleic acid 28.9 g, linolenic acid 0.3 g), and 0 mg cholesterol]. The fatty acid composition of virgin olive oil was 99.9 g fat/100 g food [14.5 g SFA (palmitic acid 14.4 g, myristic acid 0.1 g), 71 g MUFA (oleic acid 71 g), 10 g PUFA (linoleic acid 9.1 g, linolenic acid 0.9 g), and 0 mg of cholesterol].

In addition to the source of fat, breakfast also consisted of semiskimmed milk (200 mL), 18 g soluble coffee (1 monodose sachet), 8 g of sugar (1 monodose sachet), and toasted white bread (2 toasts of 10 g/u). The washing breakfast contained pineapple juice (200 mL) and peach jam (50 g), instead of butter, margarine, or virgin olive oil. The rest of the daily meals had the same nutritional composition for all the volunteers (1636  ±  527 Kcal/day, 61  ±  23 g proteins/day, 202.8  ±  58.5 g carbohydrates/day, 53,8  ±  21,8 g lipids/day, 15,1 ± 8,3 g SFA/day, 21,9 ± 9,7 g MUFA/day, 15, 1 ± 8, 3 g SFA/day, 8, 1 ± 4, 6 g PUFA/day, and 189 ± 114 mg cholesterol/day), with the exception of the type of fat chosen in the breakfast. Diet was designed based on the nutritional habits of all volunteers in order to avoid rejections. All volunteers were committed to faithfully fulfill the diet and to keep their lifestyle (physical activity, tobacco consumption, sleeping habits, meal schedules, etc.) during the study.

Experimental design included six phases: three phases of dietetic intervention in which each volunteer was randomly assigned to ingest during a month each type of breakfast and three washout periods (45 days each one). As shown in Table 1, it was verified that this washout period was sufficient to return the biochemical parameters to their baseline values.

During the selection period, weight of each subject was measured and each subject was required to fill out a questionnaire on nutritional habits and lifestyle for inclusion purposes. Each subject was also further surveyed in three occasions with 24 h dietary recalls (including three nonconsecutive days and one festive day) to realize the nutritional valuation of the habitual diet of the voluntary, using a suitable software (Dietsource v.3). Menus were designed for 30 days and they were repeated during the three months of dietetic intervention. Therefore, the type of fat consumed at the breakfast was the only nutritional variable of the study.

Due to the fact that the amount of butter, margarine, or olive oil provided was the same (20 g) for each type of breakfast, they were not isocaloric. The reason why we did not homogenize calories and the amount of fat was that the aim of the study was to find out whether a practical application could modify CDV risk factors or not.

The first and last day (pre/post) of each one of the three breakfast periods, in fasting conditions, weight, arterial blood pressure, and cardiac frequency were measured and a blood sample was taken for each volunteer.

### 2.3. Analytical Determinations

All blood extractions were performed in fasting conditions by puncture of the cubital vein. All the extractions were performed in the same conditions and samples were processed by the same staff (clinical biochemists), following the recommendations of the National for Committee Clinical Laboratory Standards [16] and using the reference values of Roche Diagnostics GmbH [17]. After centrifugation at 3000 rpm/15 minutes to obtain serum, the following determinations were performed using the analyzer Roche/Hitachi Cobas C711):* Glucose* (kitGLUC3 of Roche Diagnostics),* Urea* (kit UREAL of Roche Diagnostics),* Creatinine* (kitCREJ2 of Roche Diagnostics),* Uric acid* (kitUA2 of Roche Diagnostics),* Total protein* (kit TP2 of Roche Diagnostics),* Albumin* (kit ALB2 of Roche Diagnostics),* Cholesterol Total* (kit CHOL2 of Roche Diagnostics),* Triglycerides* (colorimetric and enzymatic test),* HDLc* (kit HDLC3 of Roche Diagnostics),* LDLc.* (Friedewald, Levy and Fredrickson formula),* AST *(*aspartate aminotransferase,* kit ASTL of Roche Diagnostics),* ALT *(*alanine aminotransferase,* kit ALTL of Roche Diagnostics), and* Sodium, Potassium, and Chloride* (ion-selective electrodes using neutral carriers for sodium and potassium determinations and ion exchangers for chloride determinations). Quality controls conducted at any moment of the study using serum controls (Roche Diagnostics) were valid and all techniques underwent satisfactory daily internal and monthly external quality controls (by Spanish Society of Clinical Chemistry), with satisfactory analytical variation (precision) and analytical deviation (exactitude). The coefficient of variation for the parameters studied ranged from 1.9 to 2.8%.

#### 2.3.1. Sample Size and Statistical Analysis

Sample size was estimated as described previously [18], with a confidence level of 95%, a power of 80%, and a twofold expected difference of the standard deviation obtained for each lipid determination. All values were expressed in International Units as mean ± SEM (standard error of the mean).

Statistical analyses were performed using SPSS statistical software (Version 17.0, SPSS Inc., Chicago, IL, USA). The Kolmogorov–Smirnov test showed agreement of the empirical distribution of the data with normality assumption. Descriptive variables were analyzed, frequency distribution table was obtained, and means of each group were compared using Student's *t*-test in conditions of normality and homoscedasticity. For other conditions, comparisons were performed using Wilcoxon's *t*-test (for paired data). In all cases, statistical significance was reached at bilateral *P* values <0.05.

## 3. Results

Results showed that recruited population was a homogeneous group of postmenopausal women (age 63.5 ± 2.5 years) with cardiovascular risk, since they were overweight or obese (BMI = 27, 8 ± 0, 6 Kg/m^2^) and they had other risk cardiovascular factors, such as CVD history, hypertension, or diabetes (Table 2).

As shown in Table 1, there were no statistical significant differences in the basal metabolic profile among the three breakfasts (A, B, and C) showing the homogeneity of the sample before breakfast ingestion and the suitability of the washout period (45 days).

**Figure 1:**
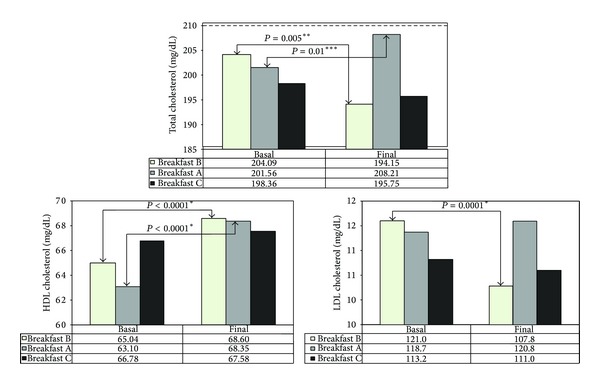
Variations in total cholesterol, HDL cholesterol, and LDL cholesterol serum concentrations with the three types of breakfast (A with butter, B with margarine, and C with olive oil). (** ): Student's *t*-test, significance level, *P* < 0.05. (*** ): Wilcoxon test, significance level, *P* < 0.05.

At the end of each treatment (daily ingestion of one type of breakfast during one month), statistical significant differences among groups were observed only in lipid profile parameters (total cholesterol and LDL) (Table 1). By comparing before and after differences for all biochemical parameters, we found out that breakfast A intake produced a statistically significant increase on total cholesterol levels (*P* = 0.01) and HDL (*P* = 0.0001), while breakfast B intake produced a statistical significant decrease on total cholesterol levels (*P* = 0.005) and LDL (*P* = 0.0001) and a concomitant statistical significant increase on HDL levels (*P* = 0.0001). Breakfast C intake did not produce any statistically significant variations in biochemical parameters. However, a tendency towards a decrease of total cholesterol and LDL levels and an increase of HDL levels was observed (Figure 1). No statistically significant differences were observed in the triglycerides concentration after the ingestion of any breakfast (data not shown).

With respect to the influence of the type of fat ingested at breakfast on cardiovascular risk parameters, no statistically significant changes were observed during the three treatment periods in BMI, neither heart rate nor arterial blood pressure (data not shown).

**Figure 2:**
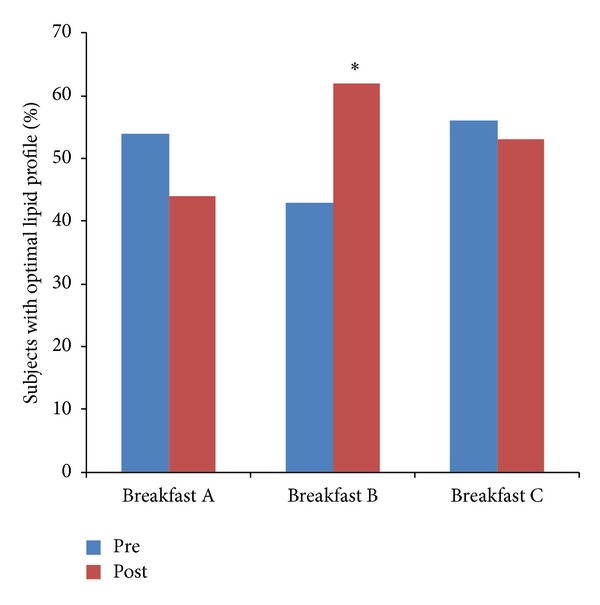
Evolution of the percentage of subjects with optimal lipid profile by comparison between baseline and final for each type of breakfast (A with butter, B with margarine and C with olive oil). (* ): *P* = 0.012.

Finally, we also studied the influence of the different breakfasts on the percentage of subjects with optimal lipid profile, defined as HDL > 35 mg/dL, LDL < 150 mg/dL, and total cholesterol < 200 mg/dL, according to NCEP-ATP III (National Cholesterol Education Program-Adult Treatment Panel III) and SEA (Sociedad Española de Arteriosclerosis) recommendations [19]. As shown in Figure 2, only the breakfast with margarine was able to produce a statistically significant increase of the percentage of subjects with optimal lipid profile.

## 4. Discussion

Studies performed to date which evaluate the effect of the different types of fats on lipid profile have been performed by analyzing the content of fat from the total daily diet of the subjects. These studies have rendered a great variety of results, which in some cases are discordant, maybe due to differences on the experimental design of these works.

In this context, the aim of this study was to evaluate whether the different types of fat ingested at breakfast are sufficient to cause variations in the plasma lipid profile of a group of women at risk of CVD. A methodological aspect to note is the fact that in our study the three types of breakfasts were not isocaloric and they did not provide the same number of grams of fat, either. In this regard, practical considerations dominated and we decided to keep the rest of breakfast components identical (and the rest of meals). Thus, the only variable was the fat source of breakfast that was provided in the most frequent form used by general population, that is, as monodose sachettes and envelopes.

Regardless of this, we think that the results obtained are relevant in terms of clinical intervention to prevent CVD. We observed that the breakfast with a polyunsaturated fat source, margarine, was able to improve the lipid profile (by decreasing cholesterol and the LDL levels and by increasing the HDL levels), while butter, as source of saturated fat in the breakfast, was only able to increase cholesterolemia at the expense of the HDL without decreasing LDL levels, the most important atherogenic agent. Moreover, it was observed that breakfast with a source of monounsaturated fat as olive oil did not modify the lipid profile of the analyzed population, maybe due to the sample size and the time of intervention or the genetic background of the subjects.

The increase in the percentage of subjects with optimal lipid profile after ingestion of breakfast containing margarine also suggests that consumption of PUFA at breakfast can be advisable in subjects at risk of CVD due to the positive effects on the lipid profile and, in consequence, decreasing the cardiovascular risk. The results obtained are also important since, according to Bray et al. [20], the type of nutrients ingested during the first meal of the day are able to program the metabolism for the rest of the day. In this regard, if high amounts of carbohydrates are ingested in the morning, they will be used more extensively for the rest of the day. If a fat-enriched breakfast is taken, the metabolism will be more flexible using both carbohydrates and fat as energy source for the rest of the day and, therefore, the fat accumulation in the adipose tissue would decrease and it would favour fat mobilization as lipoproteic cholesterol. According to our work, the mobilized fractions of lipoproteic cholesterol (HDL and LDL) could be different according to the type of fat ingested at breakfast, having PUFA a higher positive influence on lipoproteic metabolism.

Our results indicate that the substitution of saturated fat for polyunsaturated fat in the diet decreases total cholesterol levels (204.09 versus 194.15; *P* = 0.005) and LDL levels (121.02 versus 107.85; *P* = 0.0001) that could provide huge benefits to decrease the cardiovascular risk as many studies indicate [5, 21]. These results support current recommendations to modify or change the type of fat of the diet since nowadays CVD constitutes the main cause of death in developed countries. Results of the present work are in agreement with previous studies in which PUFA tend to lower plasma cholesterol levels, while saturated fatty acids tend to increase them [22, 23] and supporting that PUFA may be an ideal replacement for SFA.

In previous decades, it was thought that the use of PUFA reduced serum cholesterol and also HDL levels [24]. Mozaffarian et al. [25] also reported that PUFA lowered HDL levels and, at the same time, improved insulin resistance and reduced systematic inflammation. Results of the present study have shown the opposite, since significantly increased HDL levels were obtained (65.04 versus 68.60; *P* = 0.0001) after the intake of PUFA at breakfast, thus showing that PUFA do not always decrease HDLc levels.

On the other hand, in our study we observed that the use of monounsaturated fat in breakfast lowers total cholesterol and LDLc levels and a tendency towards an increase of HDLc, although no statistically significant differences were observed.

Pelkman et al. [26] reported that low-fat diets and monounsaturated fat-enriched diets decreased LDLc levels, although HDLc concentrations did not increase as in our work. Moreover, MUFA diets exerted favourable effects on CVD risk because the ratio of LDL to HDL improved significantly more with the MUFA diet than with either the low-fat diet or the control diet [27].

Finally, it should be noted that participants in this study had cardiovascular risk factors (overweight, arterial hypertension, diabetes, etc.), and, in this condition, the present study has shown that the fat ingested in breakfast can induce modifications on their lipid plasma profile.

## 5. Conclusion

Results of this work suggest that the use of polyunsaturated fat at breakfast is advisable in women at risk of CVD, since margarine has shown to improve the plasma lipid profile in the studied sample. It is necessary to emphasize the importance that the intake of polyunsaturated fat can have in the breakfast, as a measure of both primary and secondary prevention in women at CVD risk.

## Figures and Tables

**Table 1:** Basal and final metabolic profile before and after ingestion during 1 month of each type of breakfast.

		Breakfast with margarine		Breakfast with butter		Breakfast with olive oil		*P* (ANOVA) intertypes of breakfast	
		Basal	Final	Basal	Final	Basal	Final	Basal	Final
Glucose	Mean:	97.57	95.09	93.84	97.14	97.62	92.80	*P* > 0.05	*P* > 0.05
(mg/dL)	SEM:	1.71	1.62	1.47	1.56	2.00	1.36		

Urea	Mean:	36.83	35.17	35.96	34.82	35.82	36.80	*P* > 0.05	*P* > 0.05
(mg/dL)	SEM:	1.00	1.66	1.91	1.43	1.64	1.95		

Creatinine	Mean:	0.74	0.74	0.77	0.72	0.76	0.75	*P* > 0.05	*P* > 0.05
(mg/dL)	SEM:	0.02	0.02	0.02	0.02	0.02	0.02		

Uric acid	Mean:	4.11	4.06	4.20	4.03	4.09	4.09	*P* > 0.05	*P* > 0.05
(mg/dL)	SEM:	0.15	0.14	0.15	0.14	0.15	0.18		

Total protein	Mean:	7.40	7.44	7.42	7.48	7.51	7.34	*P* > 0.05	*P* > 0.05
(g/dL)	SEM:	0.06	0.05	0.05	0.07	0.06	0.06		

Albumin	Mean:	4.52	4.51	4.50	4.52	4.48	4.48	*P* > 0.05	*P* > 0.05
(g/dL)	SEM:	0.04	0,03	0.03	0,04	0.03	0,03		

Total cholesterol	Mean:	204.09	194.15	201.56	208.21	198.36	195.75	*P* > 0.05	**P** = 0.050
(mg/dL)	SEM:	4.25	3.65	4.71	4.67	4.45	5.19		

Triglycerides	Mean:	90.47	89.11	98.56	94.46	91.78	88.73	*P* > 0.05	*P* > 0.05
(mg/dL)	SEM:	6.34	5.24	5.64	5.69	6.28	6.10		

HDL cholesterol	Mean:	65.04	68.60	63.10	68.35	66.78	67.58	*P* > 0.05	*P* > 0.05
(mg/dL)	SEM:	1.66	1.78	1.40	1.85	1.91	1.85		

LDL cholesterol	Mean:	121.02	107.85	118.70	120.89	113.20	111.00	*P* > 0.05	**P** = 0.042
(mg/dL)	SEM:	3.75	3.10	3.85	4.01	3.66	4.76		

AST	Mean:	20.79	21.13	19.76	21.38	19.82	21.08	*P* > 0.05	*P* > 0.05
(U/L)	SEM:	0.71	0.73	0.68	0.79	0.65	1.16		

ALT	Mean:	16.77	17.91	14.88	18.81	15.73	18.95	*P* > 0.05	*P* > 0.05
(U/L)	SEM:	1.03	1.23	1.11	1.62	0.75	2.90		

Sodium	Mean:	140.98	141.36	141.64	141.14	141.11	141.20	*P* > 0.05	*P* > 0.05
(mE/L)	SEM:	0.22	0.25	0.22	0.24	0.23	0.27		

Potassium	Mean:	4.45	4.44	4.53	4.47	4.47	4.38	*P* > 0.05	*P* > 0.05
(mE/L)	SEM:	0.06	0.06	0.05	0.05	0.06	0.05		

Chloride	Mean:	104.02	104.34	104.14	103.71	103.60	104.08	*P* > 0.05	*P* > 0.05
(mE/L)	SEM:	0.28	0.31	0.26	0.28	0.34	0.34		

**Table 2:** Descriptive characteristics of the population sample.

	Means ± SEM
Age (years)	63.5 ± 2.5
Weight (kg)	63.1 ± 1.2
Height (m)	1.53 ± 0.10
BMI (kg/m^2^)	27.8 ± 0.6
SAP (mmHg)	130.35 ± 2.99
DAP (mmHg)	72.62 ± 1.32

Cardiovascular risk factors	Prevalence in the analyzed population

Arterial hypertension	27%
Diabetes mellitus	13%
Hypercholesterolemia	38%
History of CVD	17%
History of familiar CVD	32%
Overweight/obesity	73%

The data are expressed as mean ± SEM (standard error of the mean) of the 53 subjects of the study. BMI: body mass index, SAP: systolic arterial pression, DAP: diastolic arterial pression, and CVD: cardiovascular diseases.

## Abbreviations

CVD: Cardiovascular disease
BMI: Body mass index
VLDL: Very low-density lipoprotein
LDL: Low-density lipoprotein
HDL: High-density lipoprotein
SFA: Saturated fatty acids
PUFA: Poliunsaturated fatty acids
MUFA: Monounsaturated fatty acids
NCEP-ATPIII: National Cholesterol Education Program-Adult Treatment Panel III
SEA: Sociedad Española de Arteriosclerosis.
